# Mitochondrial Signaling, the Mechanisms of AKI-to-CKD Transition and Potential Treatment Targets

**DOI:** 10.3390/ijms25031518

**Published:** 2024-01-26

**Authors:** Li-Yun Chang, Yu-Lin Chao, Chien-Chih Chiu, Phang-Lang Chen, Hugo Y.-H. Lin

**Affiliations:** 1 Division of Nephrology, Department of Internal Medicine, Kaohsiung Medical University Hospital, Kaohsiung Medical University, Kaohsiung 807, Taiwan; yunchang1104@gmail.com (L.-Y.C.); leonchuo@gmail.com (Y.-L.C.); 2 Department of Biotechnology, Kaohsiung Medical University, Kaohsiung 807, Taiwan; 3 Department of Biological Chemistry, School of Medicine, University of California, Irvine, CA 92697, USA; plchen@uci.edu; 4 Division of Nephrology, Department of Internal Medicine, Kaohsiung Municipal Ta-Tung Hospital, Kaohsiung Medical University, Kaohsiung 807, Taiwan; 5 Department of Medicine, College of Medicine, Kaohsiung Medical University, Kaohsiung 807, Taiwan

**Keywords:** mitochondria, acute kidney injury, AKI, chronic kidney disease, CKD, AKT1

## Abstract

Acute kidney injury (AKI) is increasing in prevalence and causes a global health burden. AKI is associated with significant mortality and can subsequently develop into chronic kidney disease (CKD). The kidney is one of the most energy-demanding organs in the human body and has a role in active solute transport, maintenance of electrochemical gradients, and regulation of fluid balance. Renal proximal tubular cells (PTCs) are the primary segment to reabsorb and secrete various solutes and take part in AKI initiation. Mitochondria, which are enriched in PTCs, are the main source of adenosine triphosphate (ATP) in cells as generated through oxidative phosphorylation. Mitochondrial dysfunction may result in reactive oxygen species (ROS) production, impaired biogenesis, oxidative stress multiplication, and ultimately leading to cell death. Even though mitochondrial damage and malfunction have been observed in both human kidney disease and animal models of AKI and CKD, the mechanism of mitochondrial signaling in PTC for AKI-to-CKD transition remains unknown. We review the recent findings of the development of AKI-to-CKD transition with a focus on mitochondrial disorders in PTCs. We propose that mitochondrial signaling is a key mechanism of the progression of AKI to CKD and potential targeting for treatment.

## 1. Introduction

The kidneys, pivotal to maintaining health through the filtration of waste, regulation of fluids, and production of essential hormones, become a focal point when compromised, as observed in kidney disease. This compromise creates a chain reaction, triggering health and economic consequences. Kidney diseases, encompassing both acute kidney injury (AKI) and chronic kidney disease (CKD), constitute a burgeoning global burden.

AKI has emerged as a pressing concern in the global health landscape, intricately linked to a spectrum of adverse health outcomes. Its prevalence has surged over the last decade, leading to heightened morbidity and mortality rates, making it a significant global public health challenge [1,2,3,4]. In hospital settings, AKI is pervasive, affecting approximately 50% of intensive care unit (ICU) patients [5]. Despite some cases being reversible, recent evidence underscores the long-term consequences of AKI, including an elevated risk of adverse renal and cardiovascular outcomes [6,7].

Comparatively, patients experiencing AKI face a substantially increased risk of CKD development, progression of existing CKD, end stage kidney disease (ESKD), and mortality, as opposed to those with CKD without a history of AKI [8,9]. Notably, each episode of AKI doubles the risk of advanced CKD in patients with diabetes [10]. Hence, the interplay between AKI and the subsequent development of CKD carries significant clinical implications [11,12]. Understanding and addressing these intricate dynamics are paramount in pursuing effective kidney health management. 

## 2. The Intricate Relationship between AKI and CKD

Several factors can precipitate AKI, ranging from unstable hemodynamics and volume depletion to infection and exposure to nephrotoxic agents [13]. Among these, acute tubular necrosis induced by prolonged ischemia stands out as the most prevalent cause of hospital-acquired AKI [14]. Evidence increasingly underscores the complex relationship between AKI and CKD [11].

**Figure 1:**
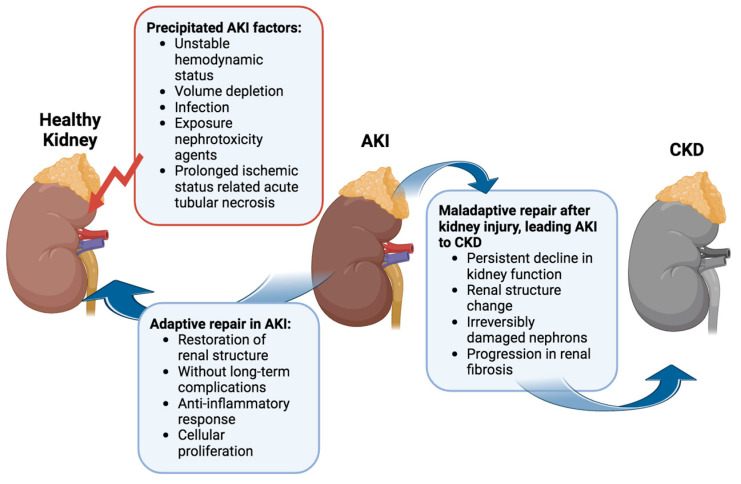
Standard perspectives on the pathophysiology in the progression from AKI to CKD: When the kidney encounters one or more precipitating factors of AKI, it can undergo repair, restoring normal or near-normal structure and function through ‘adaptive’ repair. However, maladaptive and incomplete repair can result in the development of fibrosis and, ultimately, chronic kidney disease. Figure created with biorender.com (accessed on 1 December 2023).

The progression from a transient kidney injury to sustained AKI and acute kidney disease (AKD), potentially acting as a bridge to the transition from AKI to CKD, hinges on individual susceptibility and underlying mechanisms (Figure 1) [15,16,17]. In many AKI cases, aberrant cell responses, such as abnormal cellular proliferation, sustained proinflammatory and profibrotic signaling mechanisms, progressive capillary loss, disruptions in the cell cycle, and epigenetic changes in renal cells, contribute to permanent kidney damage, culminating in renal failure [15,18,19]. Tubulointerstitial injury induces tissue hypoxia, ischemia, and vascular rarefaction, negatively impacting renal cellular function [17]. Disruptions in tubuloglomerular feedback recruit inflammatory cells and may exacerbate tubulointerstitial fibrosis. The persistent state of injury and fibrosis hinders kidney repair, leading to irreversible changes [15].

In clinical settings, the 13th Acute Dialysis Quality Initiative (ADQI) Consensus Conference differentiates between “adaptive repair” processes, facilitating the restoration of renal structure without long-term complications, and “maladaptive repair”, contributing to a persistent decline in kidney function linked to a change in renal structure [20]. The interplay between adaptive and maladaptive repair processes, alongside injury mechanisms, ultimately dictates the number of irreversibly damaged nephrons and, consequently, the long-term outlook for kidney function. Post-AKI, persistently activated fibrogenic cells drive the progression of renal fibrosis [18,21]. These factors converge, leading to the transition from AKI to CKD.

## 3. Deciphering the Pathways: From AKI Precipitants to Irreversible Nephron Damage and AKI-to-CKD Progression

### 3.1. Early Signaling Events in the Wake of Ischemia-Reperfusion Injury (IRI) 

The precise mechanisms governing the transition from AKI to CKD remain a subject of ongoing exploration. It is postulated that alterations in the intracellular and extracellular signaling of PTCs contribute significantly to the progression of this complex AKI-to-CKD transition [22,23,24].

Following an IRI, a critical phase ensues where renal blood flow is reestablished through autoregulation mechanisms mediated by the myogenic mechanism and tubuloglomerular feedback. This intricate process aims to guarantee adequate oxygen delivery for the production of essential components such as adenosine triphosphate (ATP), nitric oxide (NO), and reactive oxygen species (ROS), all integral to the homeostatic control of renal function [25,26].

In the acute aftermath of IRI, PTC releases various factors, with transforming growth factor-beta (TGF-β) and tumor necrosis factor (TNF-α), monocyte chemoattractant protein-1 (MCP-1), and transforming growth factor-beta (TGF-β) acting as key players.

**Figure 2:**
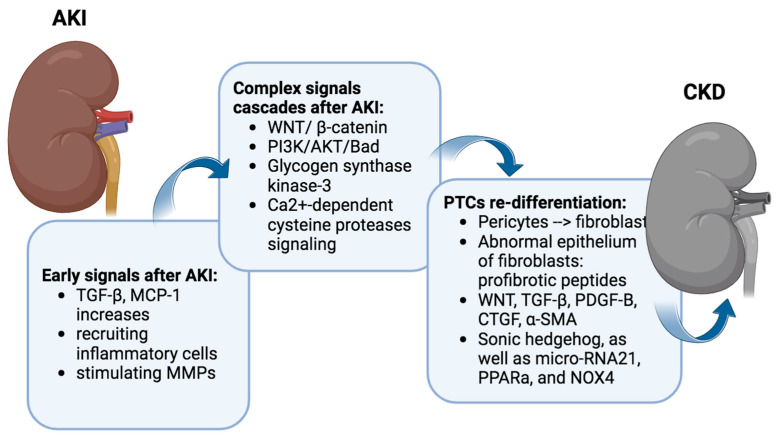
Molecular signaling in the repair/disease progression. In the early stages of ischemia-reperfusion injury (IRI), regenerating tubules release factors such as TGF-β and MCP-1, recruiting inflammatory cells and inducing cellular damage. MMP activity intensifies, leading to interstitial edema and diminished renal blood flow. Crucial signaling cascades, including WNT/β-catenin and PI3K/AKT, play key roles in the progression from AKI to CKD. WNT/β-catenin activation contributes to renal fibrotic lesions, while PI3K/AKT exhibits a potential protective role. However, inflammation, mitochondrial dysfunction, oxidative stress, and impaired nitric oxide production lead to proximal tubular cell (PTC) re-differentiation. Regenerated tubular cells transform pericytes into fibroblasts, contributing to abnormal fibroblast behavior. Diverse signaling pathways perpetuate fibroblast transformation, ultimately causing extensive kidney tissue damage. Figure created with biorender.com (accessed on 1 December 2023).

TNF-α and MCP-1 serve as chemotactic factors, orchestrating the recruitment of inflammatory cells to the site of injury. The upregulation of MCP-1 facilitates the influx of monocytes, lymphocytes, and dendritic cells into the renal tissue, contributing to the inflammatory milieu associated with IRI [27]. The upregulation of these expression may persist for up to 7 days after AKI [28]. Moreover, MCP-1 has been implicated in the modulation of various cellular processes, including oxidative stress and apoptosis, further underscoring its multifaceted role in the progression of renal damage following IRI [29]. TGF-β1, a multifunctional cytokine, exerts a dual role in the context of IRI. On one hand, it is recognized for its involvement in tissue repair and fibrosis, promoting the regeneration of damaged tubules [30]. Conversely, an excessive and dysregulated activation of TGF-β1 has been implicated in the pathogenesis of renal fibrosis and inflammation [31] (Figure 2).

### 3.2. Complex Signaling Cascades Orchestrating the Transition from AKI to CKD

IRI also instigates a complex orchestration of downstream signaling cascades, encompassing pivotal pathways such as WNT/β-catenin, PI3K/AKT/Bad, glycogen synthase kinase-3, and Ca^2+^-dependent cysteine protease signaling [32,33,34,35,36,37,38,39,40]. The Wnt/β-catenin signaling pathway plays crucial roles in organogenesis, tissue homeostasis, and the development of various diseases, including those affecting the kidneys [41]. It remains relatively quiescent in the uninjured adult kidney but undergoes reactivation during both acute and chronic renal injury. And the exaggerated activation of Wnt/β-catenin emerges as a critical player in the intricate process, contributing significantly to the developing renal fibrotic lesions marked by interstitial myofibroblast activation and excessive extracellular matrix deposition (Figure 2).

Conversely, the PI3K/AKT pathway activation displays a potential protective role, mitigating the severity of AKI [37]. However, a delicate balance in these signaling transductions is paramount, as an imbalance manifests in inflammatory cell infiltration, mitochondrial dysfunction, heightened oxidative stress, lipid peroxidation, and impaired nitric oxide production.

Post-acute inflammation, regenerated renal tubular cells dynamically engage interstitial precursor cells, orchestrating their transformation into fibroblasts, a critical step in the generation of connective tissue. Predominantly constituted by pericytes, these precursor cells intricately interact with capillaries and renal tubules [42,43,44]. Despite the regenerative efforts, certain PTCs falter in the re-differentiation process, failing to regain their regular structure [45]. These fibroblasts’ abnormal epithelium expresses profibrotic peptides and exhibits heightened signaling activity [46].

Many signaling pathways contribute to the challenge of fibroblasts failing to re-differentiate. WNT, TGF-β, Platelet-Derived Growth Factor Subunit B (PDGF-B), connective tissue growth factor (CTGF), α-smooth muscle actin (α-SMA), Sonic hedgehog, as well as micro-RNA21, PPARa, and NOX4, all play intricate roles in perpetuating this state [47,48,49,50,51,52,53,54,55,56,57]. These persistent PTC signaling pathways continue to fuel the transformation of pericytes into fibroblasts.

While these signaling and secretory responses are essential for regeneration, their timely downregulation is equally critical once tubules recover. Unfortunately, multiple activating signals disrupt this delicate equilibrium, resulting in intercellular proteolysis and cellular dissociation. At the interface of endothelial–pericyte–fibroblast interactions, pathologic events unfold, causing capillaries to rupture and fibroblasts to undergo detachment, transformation, migration, and proliferation. The consequential widening of the interstitium, brought about by proliferating fibroblasts and connective tissue, reduces capillary density [58]. The escalating transformation of pericytes into fibroblasts exacerbates kidney fibrosis, culminating in extensive kidney tissue damage. 

### 3.3. Intracellular Signaling Cascades Orchestrating the Transition from AKI to CKD

**Figure 3:**
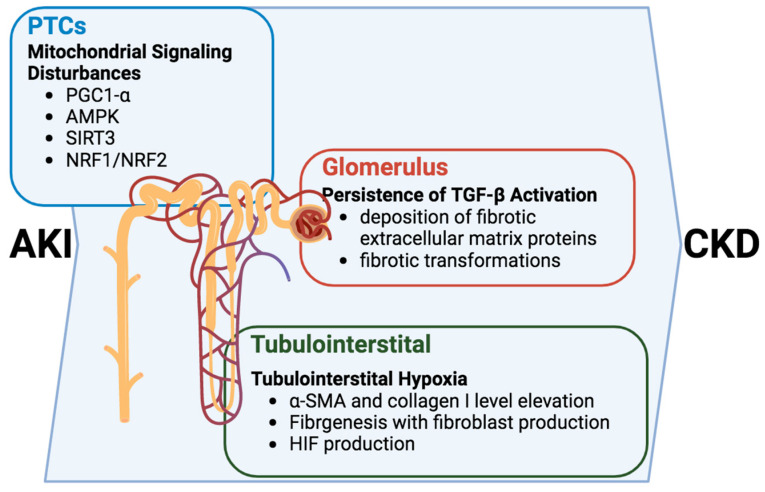
Intracellular signaling cascades orchestrating the transition from AKI to CKD: dysregulation of PGC1α, AMPK, SIRT3, and their downstream genes NRF1/NRF2 is evident in AKI. Proximal tubular cells (PTCs) are particularly affected by kidney hypoxia and mitochondrial dysfunction. Persistent TGF-β in the glomerulus induces the deposition of fibrogenic matrix proteins, leading to glomerulosclerosis. Consequently, tubulointerstitial hypoxia triggers fibrogenesis, elevating levels of α-SMA and collagen I. As the disease progresses, dysfunctional renal tubules intensify the profibrotic response, activating the secretion of TGF-β, potentially inducing the production of HIF. Figure created with biorender.com (accessed on 1 December 2023).

Alterations in intracellular signaling within renal tubular cells propel the progression of the AKI-to-CKD transition and emerge as pivotal contributors to the intricate development of tubulointerstitial fibrosis and glomerulosclerosis [22,23,24] (Figure 3). In this multifaceted cascade, the persistent activation of the TGF-β signaling pathway assumes a central role, inducing the deposition of fibrotic extracellular matrix proteins upon transitioning into the CKD phase [59,60]. The consequence of this activation is a restructuring of the renal tissue, setting the stage for the fibrotic transformations characteristic of chronic kidney disease.

PTCs play crucial roles in the tubule–glomerular interaction, and tubulointerstitial hypoxia. In tubulointerstitial hypoxia, a consequential feature operates on a dual front by triggering fibrogenesis and elevating levels of α-SMA and collagen I. This elevation catalyzes the production of fibroblasts, further contributing to the fibrotic remodeling of renal tissues [57]. While the TGF-β pathway plays a prominent role, it is crucial to recognize that the intricacies of this process involve a symphony of interconnected mechanisms.

Moreover, dysfunction within the renal tubules has a ripple effect, sensitizing tubuloglomerular feedback and intensifying the profibrotic response [61]. This heightened sensitivity, in turn, activates the secretion of TGF-β, potentially inducing the production of hypoxia-inducible factor (HIF). The induction of HIF serves as a nexus linking hypoxia, fibrogenesis, and glomerulosclerosis, adding another layer of complexity to the pathogenic progression [62].

Navigating the intricate landscape of renal pathophysiology, it is essential to recognize that the presented narrative is not all-encompassing, and there may be additional mechanisms intricately contributing to the dynamic interplay of events. Delving deeper into these complexities, further exploration is warranted to unveil the full spectrum of factors shaping the transition from acute kidney injury to chronic kidney disease. This ongoing investigation holds promise for identifying targeted therapeutic interventions and fostering a more profound comprehension of renal health. The elucidation of these intricate signaling pathways illuminates the multifaceted journey from AKI to CKD, underscoring the need for continued exploration toward refined therapeutic strategies and enhanced clinical outcomes.

## 4. Mitochondria’s Vital Role in Kidney Health

Mitochondria stand at the epicenter of cellular energy production, generating ATP and orchestrating many vital cellular functions. Their pivotal roles extend beyond mere energy provision to include the regulation of apoptosis, calcium balance, cellular differentiation, synthesizing essential macromolecules, and cellular growth [63]. Furthermore, mitochondria serve as the primary source of the cell’s reactive oxygen species (ROS) while harboring a wealth of inherent antioxidants. This is critical in maintaining the cell’s redox balance and overseeing intricate signaling pathways [63].

Structured with a double-membrane design comprising an outer membrane (OMM) that carefully regulates the selective transport of substances into and out of the mitochondria and an inner membrane (IMM) forming folds known as cristae, mitochondria house the electron transport chain. This respiratory chain, composed of five highly conserved protein complexes I–V embedded in the inner membrane, facilitates redox reactions, establishing an electrochemical gradient by concurrently transferring electrons to oxygen and transporting protons from the matrix across the inner membrane into the intermembrane space essential for cellular function. Any disruption to this delicate gradient can lead to the inability to generate ATP, impair oxidative phosphorylation, and ultimately result in the maladaptive response of PTCs following AKI [64].

The intricate architecture and functional integrity of mitochondria emerge as crucial determinants in cellular resilience, particularly in the face of physiological stressors like AKI. Understanding the nuanced interplay between mitochondrial dysfunction and AKI opens avenues for targeted interventions, promising insights that extend beyond energy metabolism to impact the broader landscape of renal health.

### 4.1. Mitochondrial Mastery: Unveiling the Distinct Needs within Kidneys

In recent years, the pivotal role of mitochondrial dysfunction has gained increasing recognition in acute and chronic renal injuries [65,66]. The kidneys, renowned for their elevated metabolic rate, receive 20% of the cardiac output, consuming 10% of the body’s oxygen intake [66,67]. This organ is notably rich in mitochondria, the energy-producing organelles crucial for maintaining cellular redox balance and energy homeostasis. Each distinct region of the kidney possesses unique energy requirements, reflected in the varying amounts of ATP they produce. For instance, in podocytes, the preservation of mitochondrial function is imperative for expressing nephrin and podocin, essential for upholding glomerular filtration [63]. Meanwhile, PTCs, the most energy-demanding cells in the kidney, are densely populated with mitochondria [64]. Impaired mitochondrial bioenergetics has the potential to interfere with vital biological processes. In addition to energy production, mitochondrial signaling pathways and the subsequent calcium flux underscore their crucial role in cellular metabolism, the generation of reactive oxygen species (ROS), and the regulation of apoptosis [65].

Mitochondria are dynamic cellular powerhouses that generate ATP through oxidative phosphorylation and play essential roles in heme biosynthesis, the Krebs cycle, fatty acid β-oxidation pathways, calcium ion homeostasis, thermogenesis, proliferation, and apoptosis [65]. The abundance of mitochondria in the kidney and susceptibility to dysfunction often associated with AKI underscores their critical regulatory role in kidney functions.

Following AKI, oxygen depletion impedes the electron transport in the mitochondrial respiratory chain, resulting in decreased ATP production. Anaerobic metabolism lowers pH and activates the Na^+^/H^+^ exchanger, prompting sodium ion influx to reduce the uptake of calcium ions by the endoplasmic reticulum (ER) [34,66,67,68,69,70,71,72]. Beyond energy depletion, mitochondria in PTCs exhibit varying degrees of swelling and fracturing post-AKI [73]. This mitochondrial damage emerges as a leading cause of cell apoptosis and necrosis in PTCs, significantly contributing to an imbalance in energy metabolism [74,75].

### 4.2. mtDNA as a Marker for Inflammation

The mitochondria have their own DNA, namely mitochondrial DNA (mtDNA), responsible for encoding essential proteins within the mitochondrial respiratory complex. It is a closed-circular double-stranded molecule housing 37 encoded genes in the mitochondrial matrix. Unlike nuclear DNA, mtDNA lacks robust repair systems and histone protection, rendering it susceptible to damage. Marked by unmethylated CpG repeats akin to bacterial DNA, mtDNA is flagged by the immune system as non-self. Post-traumatic mitochondrial damage unleashes mtDNA outside the mitochondria, particularly with low-molecular-weight cell-free mtDNA linked to inflammation and unfavorable clinical outcomes post-trauma. This released mtDNA activates inflammation through diverse signaling pathways [76,77]. In mitochondrial stress, damage, or cell death, mtDNA is released into the cellular milieu. Immune cells, such as macrophages and dendritic cells, discern the unmethylated CpG repeats in mtDNA as a danger signal [78]. This recognition sets off the Toll-like Receptor 9 (TLR9) pathway, with TLR9 binding to the CpG motifs, initiating downstream signaling cascades. Activation of TLR9 recruits adaptor proteins, such as MyD88, subsequently activating nuclear factor kappa-B (NF-κB) and mitogen-activated protein kinase (MAPK) cascades. These pathways lead to the transcription of proinflammatory cytokines like TNF-α, interleukin-1β (IL-1β), and interleukin-6 (IL-6) [79]. Additionally, mtDNA can engage other cytosolic DNA sensors, such as cyclic GMP-AMP synthase (cGAS), prompting the production of type I interferons and other inflammatory cytokines through the cGAS–STING pathway [80]. The release of these proinflammatory mediators attracts immune cells, intensifying the inflammatory response.

While this response is crucial for the body’s defense, prolonged or excessive inflammation may contribute to various pathological conditions, including AKI. Extensive population studies suggest a correlation between mtDNA copy number and renal disease, hinting at the potential use of circulating DNA copy number as a biomarker for longitudinally monitoring renal function [81]. This evolving comprehension of the intricate interplay between mitochondria and renal health illuminates the pathophysiology of kidney injuries and propels us toward innovative therapeutic strategies to preserve mitochondrial integrity and mitigate the consequences of renal dysfunction.

### 4.3. The Mitochondrial Quality Control

**Figure 4:**
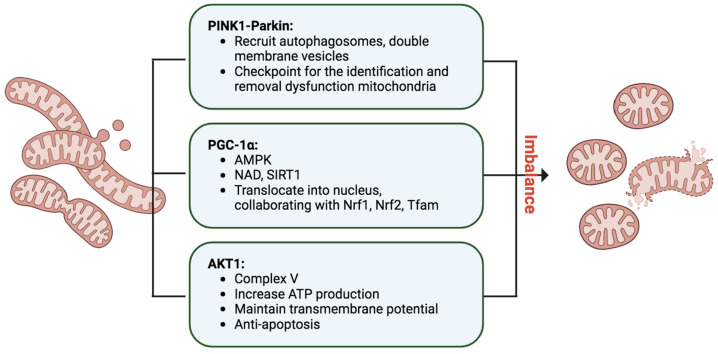
Key mitochondrial integrity control systems: the PINK1–Parkin pathway functions as a checkpoint, identifying dysfunctional mitochondria for removal. Activation of PGC1α, facilitated by AMPK and SIRT1 in response to the demand for new mitochondrial genesis, plays a crucial role. Additionally, mitochondrial AKT1 acts as a regulator in ATP production through complex V. Figure created with biorender.com (accessed on 1 December 2023).

Mitophagy, an indispensable process in cellular maintenance, meticulously oversees the removal of damaged or dysfunctional mitochondria to safeguard cellular health [82] (Figure 4). When mitochondria encounter stressors, such as oxidative damage or membrane depolarization, a tagging mechanism ensues, orchestrated by essential proteins like PINK1 and Parkin [83]. In response to mitochondrial damage, PINK1 accumulates on the outer mitochondrial membrane, activating Parkin, an E3 ubiquitin ligase. Parkin, in turn, ubiquitinates mitochondrial proteins, marking them for degradation. The recruitment of autophagosomes, double-membrane vesicles, to the ubiquitinated mitochondria facilitates their engulfment, leading to subsequent fusion with lysosomes and the formation of autolysosomes. Within these structures, damaged mitochondria undergo meticulous breakdown by lysosomal enzymes, ensuring the efficient recycling of cellular components and thwarting the accumulation of dysfunctional mitochondria. Mitophagy operates in tandem with mitochondrial biogenesis, striking a delicate balance to maintain a robust and healthy mitochondrial population within cells. This quality control mechanism is paramount for cellular homeostasis and is pivotal in mitigating conditions linked to mitochondrial dysfunction, including neurodegenerative disorders and metabolic diseases. The well-characterized PINK1–Parkin pathway exemplifies the cell’s sophisticated strategies to uphold mitochondrial integrity.

Additionally, mitochondria undergo continuous fusion and fission processes, with the PINK1–Parkin pathway serving as a checkpoint for identifying and removing defective mitochondrial segments [83]. Mitophagy, activated notably during IRI AKI, specifically targets impaired mitochondria, averting cellular mortality induced by mitochondrial oxidative stress and proapoptotic signaling. The IRI associated with AKI induces a shift in mitochondrial dynamics towards the fission process, wherein Drp1, a fission mediator, becomes phosphorylated and translocates to the mitochondria, ultimately leading to fission of the mitochondrial outer membrane [84,85,86]. This intricate interplay underscores the dynamic nature of mitophagy and its pivotal role in cellular resilience.

### 4.4. PGC-1α, the Mitochondrial Master Regulator

PGC-1α, or Peroxisome proliferator-activated receptor (PPAR)-γ coactivator-1α, assumes a pivotal role in orchestrating the intricate process of mitochondrial biogenesis, necessitating meticulous coordination between nuclear and mitochondrial genomes [87,88,89] (Figure 4). Operating as a master regulator, PGC-1α initiates a regulatory dance that propels the transcriptional machinery, increasing mitochondrial mass to meet heightened energy demands in tissues. Within the PGC-1 family, PGC-1β and PRC contribute to maintaining basal mitochondrial function and overseeing mitochondrial biogenesis in proliferating cells. The activity of PGC-1α is finely tuned through posttranslational modifications and gene expression levels, responding to stressors like glucose deprivation or exercise via AMP-activated protein kinase (AMPK)-mediated phosphorylation and activation. Elevated nicotinamide adenine dinucleotide (NAD) levels further activate Sirtuin-1 (SIRT1), a NAD-dependent deacetylase, intensifying PGC-1α activation. The active form translocates into the nucleus, collaborating with transcription factors such as Nrf1, Nrf2, and Tfam, triggering mitochondrial protein synthesis, mtDNA replication, and the genesis of new mitochondria. PGC-1α’s interactions with diverse nuclear factors influence cellular energy metabolism pathways within and outside mitochondria. While cAMP response element-binding protein (CREB) positively regulates PGC-1α transcription, inflammatory and profibrotic factors like TNF-α, TWEAK, Hes1, and TGF-β1 negatively impact PGC-1α expression, unveiling a multifaceted regulatory network. Notably, PGC-1α knockout mice exhibited compromised renal function and increased tubular injury after ischemia/reperfusion, in contrast to the improved outcomes observed in mice with PGC-1α overexpression [90]. These findings underscore PGC-1α’s protective role in mitigating kidney damage post-ischemia/reperfusion, highlighting its critical involvement in cellular energy homeostasis and potential implications for kidney disease.

### 4.5. AKT1 Shifting from Cytoplasm to Mitochondria after AKI

AKT1 kinase, also known as protein kinase B (PKB), is a serine/threonine kinase playing a crucial role in various cellular processes. Its activation is typically triggered by the binding of growth factors or other extracellular signals to cell surface receptors, initiating the activation of PI3K. PI3K generates the lipid second messenger phosphatidylinositol (3,4,5)-trisphosphate (PIP3), and AKT1 binds to PIP3 via its pleckstrin homology (PH) domain. This interaction facilitates the accessibility of T308 in the activation loop for phosphoinositide-dependent kinase 1 (PDK1). The PI3K/Akt signaling pathway stimulates cell proliferation, growth, and inhibits cell apoptosis.

Upon stimulation, AKT1 accumulates in the mitochondrial matrix, with various known substrates identified within the mitochondrion [91]. These substrates include glycogen synthesis kinase β [92], hexokinase II [93], and the β-subunit of complex V [94], indicating that AKT1 likely exerts a regulatory influence on mitochondrial processes. In our previous research, the renal tubular AKT1 was activated and translocated into mitochondria after renal IRI [95]. The intra-mitochondrial cycling of AKT1 is pivotal for modulating redox processes involved in cell cycle progression [96]. Mitochondrial Akt1 activation contributes to an anti-apoptotic effect in cardiac muscle cells [97], and dysfunction in the translocation and activation of mitochondrial AKT1 has also been linked to diabetic myocardium conditions. Furthermore, robust cardioprotection against both calcium overload and ischemic injury through the activation of mitoKATP channels depends on the associated translocation of phosphorylated AKT1 to the mitochondria [98]. It is likely that the intra-mitochondrial cycling of AKT1 after renal IRI provides a protective role. While emerging evidence supports the involvement of AKT1 in mitochondrial functions, the precise mechanism of AKT1 translocation remains poorly understood, potentially being associated with the activity of heat shock protein 90 [99].

### 4.6. Mitochondrial Iron Metabolism Dysregulation after AKI

Iron’s significant pathological role in both triggering and advancing tissue damage induced by ischemia-reperfusion (IR) is well-established. The occurrence of ferroptosis is intricately linked to the regulation of iron metabolism within the mitochondria. CISD1 (CDGSH iron sulfur domain 1), anchored in the mitochondrial outer membrane, functionally regulates mitochondrial iron absorption. Deficiency in CISD1, leading to the accumulation of mitochondrial iron and subsequent oxidative stress, has been identified as a catalyst for erastin-induced ferroptosis [100]. Furthermore, studies have revealed that cells deficient in the mitochondrial iron import protein sideroflexin 2 (SFXN2) exhibit heightened susceptibility to the ferroptosis inducer erastin. This heightened susceptibility is attributed to increased mitochondrial iron, reduced heme levels, and diminished activity of heme-dependent enzymes [101]. Catalases, peroxidases, or cytochromes P450, which rely on heme as essential cofactors, are among the affected enzymes in this context.

## 5. Critical Role of Mitochondria in AKI-to-CKD Transition

Exploring the critical nexus between AKI and the subsequent progression to CKD unveils a rich and intricate landscape of mitochondrial dynamics. This facet of research not only captivates the scientific community but also holds the promise of yielding valuable insights that could pave the way for therapeutic interventions in renal pathologies. Recent studies have notably underscored the indispensable role of mitochondrial function in orchestrating this complex transition [55,56].

CKD manifests through heightened oxidative stress, a consequence of the dysregulated production and removal of ROS [102]. Within this biochemical milieu, mitochondria emerge as central figures, assuming a dual role as victims and perpetrators. They contribute to renal damage and functional deterioration [73,74,75]. The intricacies of oxidative stress in CKD are intimately tied to impaired mitochondrial function, creating a self-perpetuating cycle that amplifies mitochondrial ROS production and, ultimately, drives cells toward apoptosis [73,74,102].

The multifaceted role of mitochondria in dictating cellular fate gains further prominence in the context of diabetic CKD. Here, renal cells succumb to mitochondrial apoptosis under the influence of elevated glucose and albumin levels, adding a layer of complexity to the interplay between metabolic factors and mitochondrial function [103]. As we delve deeper into the nuanced interconnections within this framework, it becomes increasingly apparent that understanding the role of mitochondria is paramount for deciphering the intricacies of disease progression.

This ongoing exploration into the interwoven tapestry of mitochondrial dynamics and renal health not only sheds light on the molecular mechanisms at play but also holds the potential to shape targeted interventions to mitigate the progression from AKI to CKD. Our subsequent discussion will further elucidate the multifaceted role of mitochondria in steering the trajectory of renal pathologies.

### 5.1. Lessons Learned from PGC-1α Animal Model Systems

As previously discussed, the regulatory influence of the PGC-1α factor is paramount in overseeing mitochondrial biogenesis, ensuring the synthesis of both inner and outer mitochondrial membranes, mitochondrial-encoded proteins, and the import of nuclear-encoded mitochondrial proteins, crucial for fatty acid oxidation [87,88]. In clinical settings and mouse models of AKI and CKD, there is a consistent observation of diminished PGC-1α expression [87,88,89]. This reduction corresponds with decreased mitochondrial DNA copy number, compromised membrane potential, and diminished ATP production during the transition from AKI to CKD [80,81].

Remarkably, kidney PGC-1α experiences a reduction within 24 h of kidney IRI, and PGC-1α-deficient mice exhibit more severe AKI. Conversely, the overexpression of PGC-1α in tubular cells provides protection against AKI stemming from various causes [88,89,90,104]. These findings suggest that preserving kidney PGC-1α may confer protective effects in AKI. The orchestration of mitochondrial dynamics through PGC-1α emerges as a critical determinant in the continuum of renal health. These results underscore the association between mitochondrial dysfunction and both the occurrence of AKI and the development and progression of CKD.

### 5.2. Mitochondrial Modulation in AKI: A Pathway to Prognostic Improvement

Experimental investigations have remarkably illuminated promising strategies for enhancing AKI prognosis through the modulation of mitochondrial function. Notably, instances of AKI induced by both ischemia and cisplatin showcased significant amelioration when apoptosis was strategically inhibited through the ablation of Bax and Bak [105,106]. This groundbreaking revelation highlights the intricate connection between mitochondrial dynamics and the apoptotic cascade, shedding light on potential therapeutic targets for preventing AKI progression.

Moreover, animal models simulating AKI induced by ischemia and nephrotoxicity demonstrated encouraging outcomes when mitochondrial fragmentation, a process intricately linked to cellular stress, was deliberately inhibited [107]. This underscores the pivotal role of mitochondrial structural integrity in mitigating the severity of AKI. Targeting mitochondrial dynamics is a valuable avenue for therapeutic interventions to prevent or ameliorate AKI in various clinical scenarios.

The preservation of functional mitochondria emerges as a critical factor in shielding against AKI and its subsequent renal injuries. This aligns with the growing recognition of mitochondria as energy-producing organelles and crucial cell fate and survival regulators. Mitochondrial health is intricately linked to cellular resilience, and interventions that promote mitochondrial integrity could hold the key to preventing or mitigating AKI-associated complications.

Given the acknowledged significance of tubular cell injury and death in the onset of AKI, the repair and regeneration of tubules have been identified as pivotal processes in AKI recovery [16,20,21]. This emphasizes the dynamic nature of AKI pathophysiology, where preventing initial injury and promoting regenerative processes is crucial for successful recovery. Strategies targeting tubular repair and regeneration, involving mitochondrial-focused approaches, could represent innovative directions for AKI management.

While sub-lethal injuries may exhibit reversibility, the irretrievable loss of tubular function ensues from the demise of tubular cells and intracellular mitochondrial malfunction [88]. This insight underscores the importance of preventing cell death and addressing the underlying mitochondrial dysfunction. Therapeutic interventions targeting mitochondrial repair mechanisms could provide a dual benefit by preventing cell loss and restoring cellular function.

Mitochondria are also central to the initiation of AKI, potentially paving the way for the transition to CKD following exposure to nephrotoxic agents [108]. Maleic acid (MA)-induced AKI, notably affecting renal proximal tubules, is associated with mitochondrial dysfunction [105,106]. MA-induced kidney injuries impact renal ammoniagenesis and mitochondrial energy homeostasis and induce mitochondrial permeability transition pore opening, leading to cell apoptosis [109,110,111]. Subsequent investigations reveal AKI-induced mitochondrial deformities, deficient ATP synthesis, excessive ROS accumulation, and impaired mitochondrial oxidative phosphorylation systems. Addressing mitochondrial dysfunction emerges as a critical therapeutic avenue for managing the clinical transition from AKI to CKD [75].

### 5.3. AKT1 in Focus: Unraveling Mitochondrial Signaling Dynamics in Renal Injury

Mitochondrial signaling during renal injuries remains an understudied domain; however, recent advances shed light on intricate communication pathways between mitochondria and the cytosol [91]. As we discussed earlier, several regulatory proteins linked to mitochondrial biosynthesis have been identified, including PGC-1α, AMP-activated protein kinase (AMPK), AKT, SIRT3, and Nrf1 and Nrf2 [90,91,112,113]. Of particular interest is the involvement of the PI3K/AKT signaling pathway, a fundamental intracellular pathway. The major AKT isoform expressed in PTCs, AKT1, plays a dynamic role in regulating diverse cellular functions such as angiogenesis, cell metabolism, growth/proliferation, cell survival/anti-apoptosis, protein synthesis, and gene transcription [93].

AKT1 signaling extends beyond the cytosol, demonstrating its versatility by translocating into mitochondria and the nucleus, exerting specific biological actions. For instance, insulin-stimulated AKT1 has been observed to translocate into mitochondria, influencing the oxidative phosphorylation complex V in cardiac muscle and improving bioenergetics while reducing oxidative stress in cardiomyocytes [94] (Figure 4). In the context of IRI-induced AKI, activated AKT1 was identified to translocate into PTC mitochondria [95]. Utilizing a tissue-specific, inducible overexpression animal model, mitochondrial AKT1 levels and its activation were manipulated. Inhibition of mitochondrial AKT during IRI in renal tubules resulted in exacerbated tubular damage and AKI, characterized by uncoupled mitochondrial respiration and increased oxidative stress in renal tubular epithelial cells. This scenario also led to an accelerated development of chronic kidney disease (CKD) with pronounced fibrosis.

Conversely, activating mitochondrial AKT in tubular cells during IRI protected against AKI-induced renal tubule damage and retrograde glomerulosclerosis, ultimately attenuating the progression from AKI to CKD. This animal model study strongly supports the notion of a protective role for activated mitochondrial AKT1 in renal tubule cells during IRI, preventing subsequent CKD development [95].

The intricate interplay between AKT signaling and mitochondrial dynamics in renal injury showcases the potential therapeutic relevance of targeting mitochondrial AKT to mitigate AKI and impede the subsequent development of CKD. This nuanced understanding opens avenues for interventions to preserve mitochondrial function, paving the way for novel strategies in renal health management.

In conclusion, the nexus between mitochondrial dynamics and the progression from AKI to CKD presents an intricate landscape of molecular events. Understanding these complexities opens avenues for targeted interventions to preserve mitochondrial integrity, offering hope for mitigating renal injuries and fostering kidney recovery.

## 6. Mitochondrial Targeting and Antioxidants Are the Potential Treatments for AKI and AKI-to-CKD Transition

Our comprehensive exploration has shed light on the profound involvement of mitochondrial dysfunction in the intricate pathogenesis of chronic renal disease. This dysfunction sets off a cascade of events, initiating cellular apoptosis and triggering inflammatory and fibrotic responses, thus contributing significantly to the progression of renal pathology [73].

In therapeutic interventions for renal health, promising avenues emerge, particularly in addressing maleic acid-induced nephropathy. The inhibition of the citric acid cycle following malic acid administration, as discovered by Johan Martensson in 1940, explains the observed increase in citric acid excretion after malic acid administration. When considering malic acid supplements, it is crucial to closely monitor kidney function due to its negative impact despite its potential protective effect against renal stone development. In recent years, the effects of antioxidants on renal function, especially in the context of malic acid-induced renal damage, have been extensively studied. Building on these research findings, antioxidants such as sulforaphane and curcumin have exhibited noteworthy efficacy. Their ability to mitigate redox imbalance and preserve mitochondrial bioenergetics underscores their potential as therapeutic agents in countering the adverse effects of maleic acid-induced nephropathy [114,115].

Expanding our scope to antioxidants renowned for their adeptness in scavenging ROS, including Coenzyme Q10, MitoQ, Mito-CP, and SkQR1, a compelling body of evidence supports their role in preventing AKI [107]. This highlights their relevance in the context of mitochondrial health and positions them as potential guardians against the immediate consequences of renal insult.

Taking a closer look at specific interventions targeting mitochondrial dynamics, the Szeto–Schiller peptide SS-31 emerges as a noteworthy contender. Crafted to avert the peroxidation of cardiolipin in the inner mitochondrial membrane, SS-31 can counteract mitochondrial dysfunction resulting from the accumulation of ROS, particularly in the context of an impaired oxidative phosphorylation system [116]. This peptide opens up intriguing possibilities for tailored interventions at the molecular level to safeguard mitochondrial integrity.

Furthermore, rotenone, functioning as an inhibitor of complex I in the electron transport chain, demonstrates promise in mitigating mitochondrial dysfunction. By impeding a crucial component of the energy production process, rotenone addresses the root cause of ROS accumulation, presenting a potential avenue for therapeutic intervention [117].

Expanding our repertoire of interventions, cyclosporin A emerges as a notable inhibitor of mitochondrial permeability pore formation. This intervention, coupled with the family of sirtuins, NAD+-dependent protein deacetylases [118], underscores the potential for restoring mitochondrial function post-injury. These interventions present not only a means of addressing immediate mitochondrial damage but also hold promise in preventing further cascading damage that could contribute to the progression of renal pathology [107].

## 7. Conclusions

In conclusion, mitochondrial dysfunction emerges as a pivotal contributor to the transition from AKI to CKD. The disruption of energy metabolism in PTCs during AKI manifests through abnormalities in mitochondrial biosynthesis, kinetics, signal transduction, and compromised oxidative phosphorylation. Although mitochondrial signaling can transiently compensate for fluctuations in energy supply and increased oxidative stress, persistent oxidative stress in AKI culminates in mitochondrial dysfunction, thereby contributing to the progression of CKD. The prolonged altered signal transduction between renal tubules and the glomerulus further facilitates the development of the AKI-to-CKD transition. Despite the documented significance of mitochondrial signaling in PTCs during AKI, the remote effects of mitochondrial dysfunction on the glomerulus remain unknown. This review explores recent research on the molecular pathways of mitochondrial signaling in PTCs during AKI, highlighting its role as a primary pathophysiological mechanism in AKI progression and a critical determinant of subsequent renal outcomes. Notably, targeted mitochondrial AKT1 has shown promise in reversing CKD progression, emphasizing the potential therapeutic impact of interventions to preserve mitochondrial integrity for alleviating AKI and fostering renal recovery.

In summary, the presented findings underscore the profound therapeutic potential of interventions to preserve mitochondrial integrity in mitigating AKI and promoting renal recovery. Mitochondria, once perceived solely as energy factories, are now recognized as central players in the pathophysiology of AKI. Future research and clinical endeavors should thus explore innovative strategies to safeguard mitochondrial function, offering renewed hope for enhancing AKI outcomes and ushering in a transformative era in renal care.

## Data Availability

Not applicable.

## Abbreviations

AKT1	RAC(Rho family)-alpha serine/threonine-protein kinase 1
ADQI	Acute Dialysis Quality Initiative
AKI	acute kidney injury
AMPK	AMP-activated protein kinase
ATP	adenosine triphosphate
Bad	BCL-2 antagonist of cell death
Bak	BCL-2 homologous antagonist/killer
Bax	BCL-2 activated X protein
CISD1	CDGSH iron sulfur domain 1, also known as mitoNEET
CKD	chronic kidney disease
CREB	cAMP response element-binding protein
cGAS	cyclic GMP-AMP synthase
CTGF	connective tissue growth factor
Drp1	dynamin-related protein 1
ER	endoplasmic reticulum
ESRD	end-stage renal disease
Hes1	hairy and enhancer of split-1 (a transcription factor)
HIF	hypoxia-inducible factor
IL-1b	interleukin-1β
IL-6	interleukin-6
IMM	inner membrane
IRI	ischemia-reperfusion injury
MA	maleic acid
MAPK	mitogen-activated protein kinase
MCP-1	monocyte chemoattractant protein-1
Mito-CP	mitochondria-targeted nitroxide
mitoKATP	channels mitochondrial ATP-sensitive potassium channels
MitoQ	a selective antioxidant that concentrates in the mitochondria
MMPs	matrix metalloproteinases
mtDNA	Mitochondrial DNA
MyD88	Myeloid Differentiation Primary Response Protein 88
NAD	nicotinamide adenine dinucleotide
NF-kB	nuclear factor kappa-B
NO	nitric oxide
Nox4	NADPH oxidase 4
Nrf1	nuclear respiratory factor 1
Nrf2	nuclear respiratory factor 2
OMM	outer membrane
Parkin	an E3-ubiquitin (Ub) ligase central to mitochondrial quality control
PDGF-B	Platelet-Derived Growth Factor Subunit B
PGC-1a	Peroxisome proliferator-activated receptor (PPAR)-γ coactivator-1α
PGC-1b	Peroxisome proliferator-activated receptor (PPAR)-γ coactivator-1b
PI3K	Phosphoinositide 3-kinases
PINK1	a mitochondrial kinase
PIP3	phosphatidylinositol (3,4,5)-trisphosphate
PTCs	proximal tubular cells
PRC	PGC-1-related coactivator
ROS	reactive oxygen species
SFXN2	the mitochondrial iron import protein sideroflexin 2
SIRT-1	Sirtuin-1
SIRT-3	Sirtuin-3
SKQR1	a mitochondrial targeted antioxidant
a-SMA	α-smooth muscle actin
SS-31	Szeto-Schiller peptide SS-31
TGF-b	growth factor-beta
TLR9	Toll-like Receptor 9
TNF-a	tumor necrosis factor-alpha
WNT	Wingless-related integration site, Wnt/b-catenin pathway—plays critical roles in embryonic development and adult tissue homeostasis

## References

[1] Ali T., Khan I., Simpson W., Prescott G., Townend J., Smith W., MacLeod A. (2007). Incidence and outcomes in acute kidney injury: A comprehensive population-based study. J. Am. Soc. Nephrol..

[2] Liangos O., Wald R., O’bell J.W., Price L., Pereira B.J., Jaber B.L. (2006). Epidemiology and outcomes of acute renal failure in hospitalized patients: A national survey. Clin. J. Am. Soc. Nephrol..

[3] Sawhney S., Mitchell M., Marks A., Fluck N., Black C. (2015). Long-term prognosis after acute kidney injury (AKI): What is the role of baseline kidney function and recovery? A systematic review. BMJ Open.

[4] Xue J.L., Daniels F., Star R.A., Kimmel P.L., Eggers P.W., Molitoris B.A., Himmelfarb J., Collins A.J. (2006). Incidence and mortality of acute renal failure in Medicare beneficiaries, 1992 to 2001. J. Am. Soc. Nephrol..

[5] Hoste E.A.J., Bagshaw S.M., Bellomo R., Cely C.M., Colman R., Cruz D.N., Edipidis K., Forni L.G., Gomersall C.D., Govil D. (2015). Epidemiology of acute kidney injury in critically ill patients: The multinational AKI-EPI study. Intensiv. Care Med..

[6] Anderson S., Eldadah B., Halter J.B., Hazzard W.R., Himmelfarb J., Horne F.M., Kimmel P.L., Molitoris B.A., Murthy M., O’Hare A.M. (2011). Acute kidney injury in older adults. J. Am. Soc. Nephrol..

[7] Chawla L.S., Bellomo R., Bihorac A., Goldstein S.L., Siew E.D., Bagshaw S.M., Bittleman D., Cruz D., Endre Z., Fitzgerald R.L. (2017). Acute kidney disease and renal recovery: Consensus report of the Acute Disease Quality Initiative (ADQI) 16 Workgroup. Nat. Rev. Nephrol..

[8] Coca S.G., Singanamala S., Parikh C.R. (2012). Chronic kidney disease after acute kidney injury: A systematic review and meta-analysis. Kidney Int..

[9] Wald R., Quinn R.R., Luo J., Li P., Scales D.C., Mamdani M.M., Ray J.G. (2009). Chronic dialysis and death among survivors of acute kidney injury requiring dialysis. JAMA.

[10] Thakar C.V., Christianson A., Himmelfarb J., Leonard A.C. (2011). Acute kidney injury episodes and chronic kidney disease risk in diabetes mellitus. Clin. J. Am. Soc. Nephrol..

[11] Chawla L.S., Eggers P.W., Star R.A., Kimmel P.L. (2014). Acute kidney injury and chronic kidney disease as interconnected syndromes. N. Engl. J. Med..

[12] Chawla L.S., Amdur R.L., Amodeo S., Kimmel P.L., Palant C.E. (2011). The severity of acute kidney injury predicts progression to chronic kidney disease. Kidney Int..

[13] Zuk A., Bonventre J.V. (2019). Recent advances in acute kidney injury and its consequences and impact on chronic kidney disease. Curr. Opin. Nephrol. Hypertens..

[14] Makris K., Spanou L. (2016). Acute Kidney Injury: Definition, Pathophysiology and Clinical Phenotypes. Clin. Biochem. Rev..

[15] Venkatachalam M.A., Griffin K.A., Lan R., Geng H., Saikumar P., Bidani A.K. (2010). Acute kidney injury: A springboard for progression in chronic kidney disease. Am. J. Physiol. Physiol..

[16] Basile D.P., Donohoe D., Roethe K., Osborn J.L. (2001). Renal ischemic injury results in permanent damage to peritubular capillaries and influences long-term function. Am. J. Physiol. Physiol..

[17] Basile D.P., Anderson M.D., Sutton T.A. (2012). Pathophysiology of Acute Kidney Injury. Compr. Physiol..

[18] Bechtel W., McGoohan S., Zeisberg E.M., Müller G.A., Kalbacher H., Salant D.J., Müller C.A., Kalluri R., Zeisberg M. (2010). Methylation determines fibroblast activation and fibrogenesis in the kidney. Nat. Med..

[19] Eddy A.A. (2013). The origin of scar-forming kidney myofibroblasts. Nat. Med..

[20] Basile D.P., Bonventre J.V., Mehta R., Nangaku M., Unwin R., Rosner M.H., Kellum J.A., Ronco C. (2016). Progression after AKI: Understanding Maladaptive Repair Processes to Predict and Identify Therapeutic Treatments. J. Am. Soc. Nephrol..

[21] Yang L., Besschetnova T.Y., Brooks C.R., Shah J.V., Bonventre J.V. (2010). Epithelial cell cycle arrest in G2/M mediates kidney fibrosis after injury. Nat. Med..

[22] Martin-Sanchez D., Ruiz-Andres O., Poveda J., Carrasco S., Cannata-Ortiz P., Sanchez-Niño M.D., Ortega M.R., Egido J., Linkermann A., Ortiz A. (2016). Ferroptosis, but Not Necroptosis, Is Important in Nephrotoxic Folic Acid–Induced AKI. J. Am. Soc. Nephrol..

[23] Martin-Sanchez D., Fontecha-Barriuso M., Carrasco S., Sanchez-Niño M.D., von Mässenhausen A., Linkermann A., Cannata-Ortiz P., Ruiz-Ortega M., Egido J., Ortiz A. (2018). TWEAK and RIPK1 mediate a second wave of cell death during AKI. Proc. Natl. Acad. Sci. USA.

[24] Kaushal G.P., Shah S.V. (2016). Autophagy in acute kidney injury. Kidney Int..

[25] Aksu U., Demirci C., Ince C. (2011). The pathogenesis of acute kidney injury and the toxic triangle of oxygen, reactive oxygen species and nitric oxide. Contrib Nephrol..

[26] Just A. (2007). Mechanisms of renal blood flow autoregulation: Dynamics and contributions. Am. J. Physiol. Integr. Comp. Physiol..

[27] Cantero-Navarro E., Rayego-Mateos S., Orejudo M., Tejedor-Santamaria L., Tejera-Muñoz A., Sanz A.B., Marquez-Exposito L., Marchant V., Santos-Sanchez L., Egido J. (2021). Role of Macrophages and Related Cytokines in Kidney Disease. Front. Med..

[28] Rodríguez-Romo R., Berman N., Gómez A., Bobadilla N.A. (2015). Epigenetic regulation in the acute kidney injury to chronic kidney disease transition. Nephrology.

[29] Singh S., Anshita D., Ravichandiran V. (2021). MCP-1: Function, regulation, and involvement in disease. Int. Immunopharmacol..

[30] Lodyga M., Hinz B. (2020). TGF-beta1—A truly transforming growth factor in fibrosis and immunity. Semin. Cell Dev. Biol..

[31] Loboda A., Sobczak M., Jozkowicz A., Dulak J. (2016). TGF-beta1/Smads and miR-21 in Renal Fibrosis and Inflammation. Mediators Inflamm.

[32] Rabb H., Griffin M.D., McKay D.B., Swaminathan S., Pickkers P., Rosner M.H., Kellum J.A., Ronco C. (2016). Inflammation in AKI: Current Understanding, Key Questions, and Knowledge Gaps. J. Am. Soc. Nephrol..

[33] Isaac J., Tögel F.E., Westenfelder C. (2007). Extent of glomerular tubularization is an indicator of the severity of experimental acute kidney injury in mice. Nephron Exp. Nephrol..

[34] Rodríguez D., Morrison C.J., Overall C.M. (2010). Matrix metalloproteinases: What do they not do? New substrates and biological roles identified by murine models and proteomics. Biochim. Biophys. Acta (BBA)-Mol. Cell Res..

[35] Novak K.B., Le H.D., Christison-Lagay E.R., Nose V., Doiron R.J., Moses M.A., Puder M. (2010). Effects of metalloproteinase inhibition in a murine model of renal ischemia-reperfusion injury. Pediatr. Res..

[36] Zhang G., Wang Q., Zhou Q., Wang R., Xu M., Wang H., Wang L., Wilcox C.S., Liu R., Lai E.Y. (2016). Protective Effect of Tempol on Acute Kidney Injury Through PI3K/Akt/Nrf2 Signaling Pathway. Kidney Blood Press. Res..

[37] Liu C., Chen K., Wang H., Zhang Y., Duan X., Xue Y., He H., Huang Y., Chen Z., Ren H. (2020). Gastrin Attenuates Renal Ischemia/Reperfusion Injury by a PI3K/Akt/Bad-Mediated Anti-apoptosis Signaling. Front. Pharmacol..

[38] Jamadar A., Rao R. (2020). Glycogen Synthase Kinase-3 Signaling in Acute Kidney Injury. Nephron.

[39] Bonventre J.V., Yang L. (2011). Cellular pathophysiology of ischemic acute kidney injury. J. Clin. Investig..

[40] Zhang D.-Y., Wang H.-J., Tan Y.-Z. (2011). Wnt/beta-catenin signaling induces the aging of mesenchymal stem cells through the DNA damage response and the p53/p21 pathway. PLoS ONE.

[41] Hong X., Zhou Y., Wang D., Lyu F., Guan T., Liu Y., Xiao L. (2021). Exogenous Wnt1 Prevents Acute Kidney Injury and Its Subsequent Progression to Chronic Kidney Disease. Front. Physiol..

[42] Humphreys B.D., Lin S.-L., Kobayashi A., Hudson T.E., Nowlin B.T., Bonventre J.V., Valerius M.T., McMahon A.P., Duffield J.S. (2010). Fate tracing reveals the pericyte and not epithelial origin of myofibroblasts in kidney fibrosis. Am. J. Pathol..

[43] Duffield J.S. (2014). Cellular and molecular mechanisms in kidney fibrosis. J. Clin. Investig..

[44] Picard N., Baum O., Vogetseder A., Kaissling B., Le Hir M. (2008). Origin of renal myofibroblasts in the model of unilateral ureter obstruction in the rat. Histochem. Cell Biol..

[45] Humphreys B.D., Czerniak S., DiRocco D.P., Hasnain W., Cheema R., Bonventre J.V. (2011). Repair of injured proximal tubule does not involve specialized progenitors. Proc. Natl. Acad. Sci. USA.

[46] Lan R., Geng H., Polichnowski A.J., Singha P.K., Saikumar P., McEwen D.G., Griffin K.A., Koesters R., Weinberg J.M., Bidani A.K. (2012). PTEN loss defines a TGF-β-induced tubule phenotype of failed differentiation and JNK signaling during renal fibrosis. Am. J. Physiol. Physiol..

[47] Lin S.-L., Chang F.-C., Schrimpf C., Chen Y.-T., Wu C.-F., Wu V.-C., Chiang W.-C., Kuhnert F., Kuo C.J., Chen Y.-M. (2011). Targeting endothelium-pericyte cross talk by inhibiting VEGF receptor signaling attenuates kidney microvascular rarefaction and fibrosis. Am. J. Pathol..

[48] Chen Y.-T., Chang F.-C., Wu C.-F., Chou Y.-H., Hsu H.-L., Chiang W.-C., Shen J., Chen Y.-M., Wu K.-D., Tsai T.-J. (2011). Platelet-derived growth factor receptor signaling activates pericyte–myofibroblast transition in obstructive and post-ischemic kidney fibrosis. Kidney Int..

[49] Wu C.-F., Chiang W.-C., Lai C.-F., Chang F.-C., Chen Y.-T., Chou Y.-H., Wu T.-H., Linn G.R., Ling H., Wu K.-D. (2013). Transforming growth factor β-1 stimulates profibrotic epithelial signaling to activate pericyte-myofibroblast transition in obstructive kidney fibrosis. Am. J. Pathol..

[50] Ren S., Johnson B.G., Kida Y., Ip C., Davidson K.C., Lin S.-L., Kobayashi A., Lang R.A., Hadjantonakis A.-K., Moon R.T. (2013). LRP-6 is a coreceptor for multiple fibrogenic signaling pathways in pericytes and myofibroblasts that are inhibited by DKK-1. Proc. Natl. Acad. Sci. USA.

[51] Chau B.N., Xin C., Hartner J., Ren S., Castano A.P., Linn G., Li J., Tran P.T., Kaimal V., Huang X. (2012). MicroRNA-21 promotes fibrosis of the kidney by silencing metabolic pathways. Sci. Transl. Med..

[52] Li S., Mariappan N., Megyesi J., Shank B., Kannan K., Theus S., Price P.M., Duffield J.S., Portilla D. (2013). Proximal tubule PPARα attenuates renal fibrosis and inflammation caused by unilateral ureteral obstruction. Am. J. Physiol. Physiol..

[53] DiRocco D.P., Kobayashi A., Taketo M.M., McMahon A.P., Humphreys B.D. (2013). Wnt4/β-catenin signaling in medullary kidney myofibroblasts. J. Am. Soc. Nephrol..

[54] Zhou D., Li Y., Zhou L., Tan R.J., Xiao L., Liang M., Hou F.F., Liu Y. (2014). Sonic hedgehog is a novel tubule-derived growth factor for interstitial fibroblasts after kidney injury. J. Am. Soc. Nephrol..

[55] Barnes J.L., Gorin Y. (2011). Myofibroblast differentiation during fibrosis: Role of NAD(P)H oxidases. Kidney Int..

[56] Eardley K.S., Kubal C., Zehnder D., Quinkler M., Lepenies J., Savage C.O., Howie A.J., Kaur K., Cooper M.S., Adu D. (2008). The role of capillary density, macrophage infiltration and interstitial scarring in the pathogenesis of human chronic kidney disease. Kidney Int..

[57] Palm F., Nordquist L. (2011). Renal tubulointerstitial hypoxia: Cause and consequence of kidney dysfunction. Clin. Exp. Pharmacol. Physiol..

[58] Fligny C., Duffield J.S. (2013). Activation of pericytes: Recent insights into kidney fibrosis and microvascular rarefaction. Curr. Opin. Rheumatol..

[59] Liu R., Layton A.T. (2016). Modeling the effects of positive and negative feedback in kidney blood flow control. Math. Biosci..

[60] Sun D., Samuelson L.C., Yang T., Huang Y., Paliege A., Saunders T., Briggs J., Schnermann J. (2001). Mediation of tubuloglomerular feedback by adenosine: Evidence from mice lacking adenosine 1 receptors. Proc. Natl. Acad. Sci. USA.

[61] Araujo M., Welch W.J., Monu S.R., Maheshwari M., Peterson E.L., Carretero O.A., Zhou X., Sullivan K., Walsh S., Pasternak A. (2009). Cyclooxygenase 2 inhibition suppresses tubuloglomerular feedback: Roles of thromboxane receptors and nitric oxide. Am. J. Physiol. Physiol..

[62] Hanna C., Hubchak S.C., Liang X., Rozen-Zvi B., Schumacker P.T., Hayashida T., Schnaper H.W., Zhao Y., Zeng H., Liu B. (2013). Hypoxia-inducible factor-2α and TGF-β signaling interact to promote normoxic glomerular fibrogenesis. Am. J. Physiol. Physiol..

[63] Chan D.C. (2006). Mitochondria: Dynamic Organelles in Disease, Aging, and Development. Cell.

[64] Maekawa H., Inagi R. (2019). Pathophysiological Role of Organelle Stress/Crosstalk in AKI-to-CKD Transition. Semin. Nephrol..

[65] Nunnari J., Suomalainen A. (2012). Mitochondria: In Sickness and in Health. Cell.

[66] Che R., Yuan Y., Huang S., Zhang A. (2014). Mitochondrial dysfunction in the pathophysiology of renal diseases. Am. J. Physiol. Renal. Physiol..

[67] Duann P., Lin P.H. (2017). Mitochondria Damage and Kidney Disease. Adv. Exp. Med. Biol..

[68] Bhargava P., Schnellmann R.G. (2017). Mitochondrial energetics in the kidney. Nat. Rev. Nephrol..

[69] Kim E.Y., Anderson M., Dryer S.E. (2012). Sustained activation of N-methyl-D-aspartate receptors in podoctyes leads to oxidative stress, mobilization of transient receptor potential canonical 6 channels, nuclear factor of activated T cells activation, and apoptotic cell death. Mol. Pharmacol..

[70] Kaushal G.P., Kaushal V., Herzog C., Yang C. (2008). Autophagy delays apoptosis in renal tubular epithelial cells in cisplatin cytotoxicity. Autophagy.

[71] Nath K.A. (1992). Tubulointerstitial changes as a major determinant in the progression of renal damage. Am. J. Kidney Dis..

[72] Isaka Y., Kimura T., Takabatake Y. (2011). The protective role of autophagy against aging and acute ischemic injury in kidney proximal tubular cells. Autophagy.

[73] Brooks C., Wei Q., Cho S.-G., Dong Z. (2009). Regulation of mitochondrial dynamics in acute kidney injury in cell culture and rodent models. J. Clin. Investig..

[74] Sun J., Zhang J., Tian J., Virzì G.M., Digvijay K., Cueto L., Yin Y., Rosner M.H., Ronco C. (2019). Mitochondria in Sepsis-Induced AKI. J. Am. Soc. Nephrol..

[75] Jiang M., Bai M., Lei J., Xie Y., Xu S., Jia Z., Zhang A. (2020). Mitochondrial dysfunction and the AKI-to-CKD transition. Am. J. Physiol. Physiol..

[76] Riley J.S., Tait S.W. (2020). Mitochondrial DNA in inflammation and immunity. Embo Rep..

[77] Shi L., Zha H., Pan Z., Wang J., Xia Y., Li H., Huang H., Yue R., Song Z., Zhu J. (2023). DUSP1 protects against ischemic acute kidney injury through stabilizing mtDNA via interaction with JNK. Cell Death Dis..

[78] Bao W., Xia H., Liang Y., Ye Y., Lu Y., Xu X., Duan A., He J., Chen Z., Wu Y. (2016). Toll-like Receptor 9 Can be Activated by Endogenous Mitochondrial DNA to Induce Podocyte Apoptosis. Sci. Rep..

[79] Ahmad S. (2007). Turning on TLR9. Nat. Rev. Immunol..

[80] Mankan A.K., Schmidt T., Chauhan D., Goldeck M., Honing K., Gaidt M., Kubarenko A.V., Andreeva L., Hopfner K.P., Hornung V. (2014). Cytosolic RNA:DNA hybrids activate the cGAS-STING axis. EMBO J..

[81] Malik A.N. (2023). Mitochondrial DNA—Novel mechanisms of kidney damage and potential biomarker. Curr. Opin. Nephrol. Hypertens..

[82] Westermann B. (2010). Mitochondrial fusion and fission in cell life and death. Nat. Rev. Mol. Cell Biol..

[83] Pickrell A.M., Youle R.J. (2015). The roles of PINK1, parkin, and mitochondrial fidelity in Parkinson’s disease. Neuron.

[84] Li H., Feng J., Zhang Y., Feng J., Wang Q., Zhao S., Meng P., Li J. (2019). Mst1 deletion attenuates renal ischaemia-reperfusion injury: The role of microtubule cytoskeleton dynamics, mitochondrial fission and the GSK3β-p53 signalling pathway. Redox Biol..

[85] Wang Y., Liu Q., Cai J., Wu P., Wang D., Shi Y., Huyan T., Su J., Li X., Wang Q. (2022). Emodin prevents renal ischemia-reperfusion injury via suppression of CAMKII/DRP1-mediated mitochondrial fission. Eur. J. Pharmacol..

[86] Zheng Q.-Y., Li Y., Liang S.-J., Chen X.-M., Tang M., Rao Z.-S., Li G.-Q., Feng J.-L., Zhong Y., Chen J. (2022). LIGHT deficiency attenuates acute kidney disease development in an in vivo experimental renal ischemia and reperfusion injury model. Cell Death Discov..

[87] Li S.-Y., Susztak K. (2018). The Role of Peroxisome Proliferator-Activated Receptor γ Coactivator 1α (PGC-1α) in Kidney Disease. Semin. Nephrol..

[88] Portilla D., Dai G., McClure T., Bates L., Kurten R., Megyesi J., Price P., Li S. (2002). Alterations of PPARα and its coactivator PGC-1 in cisplatin-induced acute renal failure. Kidney Int..

[89] Tran M., Tam D., Bardia A., Bhasin M., Rowe G.C., Kher A., Zsengeller Z.K., Akhavan-Sharif M.R., Khankin E.V., Saintgeniez M. (2011). PGC-1α promotes recovery after acute kidney injury during systemic inflammation in mice. J. Clin. Investig..

[90] Han S.H., Wu M.-Y., Nam B.Y., Park J.T., Yoo T.-H., Kang S.-W., Park J., Chinga F., Li S.-Y., Susztak K. (2017). PGC-1α Protects from Notch-Induced Kidney Fibrosis Development. J. Am. Soc. Nephrol..

[91] Bijur G.N., Jope R.S. (2003). Rapid accumulation of Akt in mitochondria following phosphatidylinositol 3-kinase activation. J. Neurochem..

[92] Linseman D.A., Butts B.D., Precht T.A., Phelps R.A., Le S.S., Laessig T.A., Bouchard R.J., Florez-McClure M.L., Heidenreich K.A. (2004). Glycogen synthase kinase-3beta phosphorylates Bax and promotes its mitochondrial localization during neuronal apoptosis. J. Neurosci..

[93] Roberts D.J., Miyamoto S. (2015). Hexokinase II integrates energy metabolism and cellular protection: Akting on mitochondria and TORCing to autophagy. Cell Death Differ..

[94] Yang J.-Y., Deng W., Chen Y., Fan W., Baldwin K.M., Jope R.S., Wallace D.C., Wang P.H. (2013). Impaired translocation and activation of mitochondrial Akt1 mitigated mitochondrial oxidative phosphorylation Complex V activity in diabetic myocardium. J. Mol. Cell. Cardiol..

[95] Lin H.Y.-H., Chen Y., Chen Y.-H., Ta A.P., Lee H.-C., MacGregor G.R., Vaziri N.D., Wang P.H. (2020). Tubular mitochondrial AKT1 is activated during ischemia reperfusion injury and has a critical role in predisposition to chronic kidney disease. Kidney Int..

[96] Arciuch V.G.A., Galli S., Franco M.C., Lam P.Y., Cadenas E., Carreras M.C., Poderoso J.J. (2009). Akt1 intramitochondrial cycling is a crucial step in the redox modulation of cell cycle progression. PLoS ONE.

[97] Su C.-C., Yang J.-Y., Leu H.-B., Chen Y., Wang P.H. (2012). Mitochondrial Akt-regulated mitochondrial apoptosis signaling in cardiac muscle cells. Am. J. Physiol. Circ. Physiol..

[98] Ahmad N., Wang Y., Haider K.H., Wang B., Pasha Z., Uzun O., Ashraf M. (2006). Cardiac protection by mitoK_ATP_ channels is dependent on Akt translocation from cytosol to mitochondria during late preconditioning. Am. J. Physiol. Circ. Physiol..

[99] Barksdale K.A., Bijur G.N. (2009). The basal flux of Akt in the mitochondria is mediated by heat shock protein 90. J. Neurochem..

[100] Yuan H., Li X., Zhang X., Kang R., Tang D. (2016). CISD1 inhibits ferroptosis by protection against mitochondrial lipid peroxidation. Biochem. Biophys. Res. Commun..

[101] Chen Y., Qian J., Ding P., Wang W., Li X., Tang X., Tang C., Yang Y., Gu C. (2022). Elevated SFXN2 limits mitochondrial autophagy and increases iron-mediated energy production to promote multiple myeloma cell proliferation. Cell Death Dis..

[102] Granata S., Gassa A.D., Tomei P., Lupo A., Zaza G. (2015). Mitochondria: A new therapeutic target in chronic kidney disease. Nutr. Metab..

[103] Ho H.-J., Shirakawa H. (2022). Oxidative Stress and Mitochondrial Dysfunction in Chronic Kidney Disease. Cells.

[104] Tran M.T., Zsengeller Z.K., Berg A.H., Khankin E.V., Bhasin M.K., Kim W., Clish C.B., Stillman I.E., Karumanchi S.A., Rhee E.P. (2016). PGC1α drives NAD biosynthesis linking oxidative metabolism to renal protection. Nature.

[105] Wei Q., Dong G., Chen J.-K., Ramesh G., Dong Z. (2013). Bax and Bak have critical roles in ischemic acute kidney injury in global and proximal tubule–specific knockout mouse models. Kidney Int..

[106] Alassaf N., Attia H. (2023). Autophagy and necroptosis in cisplatin-induced acute kidney injury: Recent advances regarding their role and therapeutic potential. Front. Pharmacol..

[107] Li Y., Hepokoski M., Gu W., Simonson T., Singh P. (2021). Targeting Mitochondria and Metabolism in Acute Kidney Injury. J. Clin. Med..

[108] Roginski A.C., Zemniaçak B., Marschner R.A., Wajner S.M., Ribeiro R.T., Wajner M., Amaral A.U. (2022). Disruption of mitochondrial functions involving mitochondrial permeability transition pore opening caused by maleic acid in rat kidney. J. Bioenerg. Biomembr..

[109] Lin H.Y.-H., Liang C.-J., Liu M.-C., Huang M.-F., Chang J.-S., Liang S.-S. (2018). The use of chemical probes to detect the proteomics of renal tubular injury induced by maleic acid. J. Chromatogr. A.

[110] Zager R.A., Johnson A.C.M., Naito M., Bomsztyk K. (2008). Maleate nephrotoxicity: Mechanisms of injury and correlates with ischemic/hypoxic tubular cell death. Am. J. Physiol. Physiol..

[111] Roginski A.C., Cecatto C., Wajner S.M., Camera F.D., Castilho R.F., Wajner M., Amaral A.U. (2019). Experimental evidence that maleic acid markedly compromises glutamate oxidation through inhibition of glutamate dehydrogenase and α-ketoglutarate dehydrogenase activities in kidney of developing rats. Mol. Cell. Biochem..

[112] Fontecha-Barriuso M., Martin-Sanchez D., Martinez-Moreno J.M., Monsalve M., Ramos A.M., Sanchez-Niño M.D., Ruiz-Ortega M., Ortiz A., Sanz A.B. (2020). The Role of PGC-1α and Mitochondrial Biogenesis in Kidney Diseases. Biomolecules.

[113] Benigni A., Perico L., Macconi D., Poljsak B., Milisav I., Zou X., Santa-Maria C.A., O’Brien J., Gius D., Zhu Y. (2016). Mitochondrial Dynamics Is Linked to Longevity and Protects from End-Organ Injury: The Emerging Role of Sirtuin 3. Antioxid. Redox Signal..

[114] Tapia E., Sanchez-Lozada L.-G., García-Niño W.R., García F.E., Cerecedo A., García-Arroyo F.E., Osorio H., Arellano A., Cristobal-Garcia M., Loredo M.L. (2014). Curcumin prevents maleate-induced nephrotoxicity: Relation to hemodynamic alterations, oxidative stress, mitochondrial oxygen consumption and activity of respiratory complex I. Free Radic. Res..

[115] Briones-Herrera A., Avila-Rojas S.H., Aparicio-Trejo O.E., Cristóbal M., León-Contreras J.C., Hernández-Pando R., Pinzón E., Pedraza-Chaverri J., Sánchez-Lozada L.G., Tapia E. (2018). Sulforaphane prevents maleic acid-induced nephropathy by modulating renal hemodynamics, mitochondrial bioenergetics and oxidative stress. Food Chem. Toxicol..

[116] Birk A.V., Liu S., Soong Y., Mills W., Singh P., Warren J.D., Seshan S.V., Pardee J.D., Szeto H.H. (2013). The mitochondrial-targeted compound SS-31 re-energizes ischemic mitochondria by interacting with cardiolipin. J. Am. Soc. Nephrol..

[117] Sun Y., Zhang Y., Zhao D., Ding G., Huang S., Zhang A., Jia Z. (2014). Rotenone remarkably attenuates oxidative stress, inflammation, and fibrosis in chronic obstructive uropathy. Mediat. Inflamm..

[118] Morigi M., Perico L., Benigni A. (2018). Sirtuins in Renal Health and Disease. J. Am. Soc. Nephrol..

